# Practitioner perspectives on the child and youth mental health system in Australia: what needs to change?

**DOI:** 10.1080/00049530.2025.2509649

**Published:** 2025-06-04

**Authors:** Janice M. Kan, Trisha Nowland, Lucy A. Tully, Olivia Liew, Lena Gibbons, David J. Hawes, Mark R. Dadds

**Affiliations:** a School of Psychology, University of Sydney, Sydney, Australia; b School of Psychological Sciences, College of Health and Medicine, University of Tasmania, Hobart, Australia; c School of Psychological Sciences, Macquarie University, Sydney, Australia; d Growing Minds Australia, Sydney, Australia

**Keywords:** Children, young people, survey, mental health, health service

## Abstract

**Objective:**

Mental health problems in children and young people in Australia are not improving and continue to have a costly impact. Significant changes to the current child youth mental health (CYMH) system are needed and should be informed by key stakeholders such as practitioners “on the ground” delivering services.

**Method:**

Australian practitioners (*N* = 206) working in CYMH were surveyed using quantitative rating scales and qualitative open-response items. The survey examined current waitlists and waitlist management strategies, treatment dropouts, perceived barriers to accessing care, satisfaction levels, and ease of navigating services.

**Results:**

Around 70% practitioners reported being dissatisfied with the current CYMH system in meeting the needs of children, young people, and families. About 50% reported it is difficult to navigate the system to find referrals. About half of practitioners reported their service currently has a waitlist. Of those practitioners, approximately 32% reported wait times of between one and three months, and 21% reported wait times of three-to-six months. Qualitative responses from practitioners highlighted several systems issues including long waitlists, underfunded public services, and a lack of specialised training in CYMH.

**Conclusion:**

The results reveal several practice, policy, and research priorities for improving the CYMH system in Australia.

## KEY POINTS

**What is already known about this topic:**

1. Like what is observed globally, the prevalence of mental health disorders in children and young people in Australia is not decreasing.
2. Key stakeholders in the Australian mental health system are well placed to provide commentary on issues and targets for change in the current system, though most existing studies focus on parents.
3. There is limited research exploring practitioner perspectives about the current child youth mental health system.

**What this topic adds:**

1. This study provides a recent account of the state of the child youth mental health system on issues such as waitlists, approaches to managing treatment drop-outs, and satisfaction levels.
2. The majority of surveyed practitioners were dissatisfied with the current system, and their perceived barriers for accessing care were similar to those previously reported by parents.
3. Several priorities of change were identified from the results, including increased government funding, enhanced workforce training based on core child youth mental health competencies, and further research into technology-enhanced service delivery.

## Introduction

Despite Australia’s relative wealth, investment in mental health services, and increasing recognition of the importance of early intervention, the prevalence of child youth mental health (CYMH) disorders is not decreasing (Sawyer et al., 2018). Findings from the most recent Australian Child and Adolescent Survey of Mental Health and Wellbeing highlights mental illness as the most common and burdensome health condition in children and adolescents. Almost 1 in 7 children and young people aged 4–17 years experienced mental illness in the previous 12 months (Lawrence et al., 2016), and these findings parallel those found globally, where rates of mental health diagnoses and impact on functioning have increased in children and adolescents (Piao et al., 2022). In Australia, just over half of children and young people in need accessed mental health and support services, and those whose needs were not fully met endorsed a number of barriers, including not being sure where to get help, not being able to afford help, and preferring to handle the problem by themselves (Lawrence et al., 2016). Evidently, strategies are needed to improve the current mental health system and better meet the needs of children, young people, and families. To determine specific targets for change, the perspectives of multiple stakeholders involved are needed.

Much of the research highlighting problems in the system and possible contributing factors has involved parent’s perspectives (Boulter & Rickwood, 2013; Johnson et al., 2018; Lawrence et al., 2016; Reardon et al., 2017). Practitioners who deliver CYMH and wellbeing services “on the ground” are relatively understudied, despite being a critical stakeholder and key source of information regarding the current system. To our knowledge, only three studies utilising qualitative interviews from one research group have explored the perspectives of Australian practitioners working in CYMH. One nation-wide study interviewed psychologists, psychiatrists, and paediatricians about barriers and enablers to mental health care for children with Autism Spectrum Disorder (ASD) and Attention-Deficit-Hyperactivity Disorder (ADHD; Paton et al., 2019). Subsequent studies by this group interviewed the same type of clinicians and also included GPs in Victoria and South Australia only, focusing on access to care for children and adolescents with mild-to-moderate ADHD and anxiety (Paton et al., 2021), and the role of the education system in supporting mental health care (Paton et al., 2022). Results highlighted the important role of educators in supporting the paediatric mental health system, and factors such as poor mental health literacy, restricted access to services, and limited specialist support and training, are key problems in the CYMH system.

While previous studies surveying practitioners revealed important information about issues and enablers of change, they did not include the perspectives of a broad range of professionals who also provide mental health care in Australia, including social workers, nurses, and case workers. Practitioners from different professional backgrounds are important to include to obtain rich, varied, and additional insights about factors impacting the current mental health system, such as service navigation and barriers to care, which may differ depending on the service context and nature of the specific discipline. Further, questions were not asked about specific issues and practices such as waitlists, waitlist management, and drop-outs, which have been shown to impact client and practitioner outcomes. Increased wait times can lead to deterioration in client outcomes (Reichert & Jacobs, 2018) and predict treatment drop-out once clients access care (Carter et al., 2012), and managing waitlists can create high emotional burden for practitioners (McDonnell et al., 2022). In addition, untreated mental health problems (whether through difficulties accessing care or early termination) in children and young people are associated with long-term mental health problems and impacts to functioning in adulthood (Kessler et al., 2007). While research suggests waitlists and treatment drop-outs are indicators of mental health system functioning, there is limited current data on these factors in the Australian CYMH system. This is the first Australian study of practitioners using mixed methods to gather data nation-wide on CYMH system including a range of issues that were not specifically targeted in previous studies. Our aim was to recruit practitioners from a range of professions providing CYMH services to examine: 1) current waitlists and waitlist management strategies, 2) approaches to managing dropouts, 3) perceived barriers for children, youth and families accessing timely care; 4) and current practitioner satisfaction with the CYMH system, including their perceived ease of navigating CYMH services for clients.

## Method

### Procedure

The Human Research Ethics Committee at the University of Sydney provided ethics approval for the study (approval number: 2022/337). The survey was anonymous and took approximately 20 min to complete. Participants were invited to go into a draw to win one of five gift vouchers.

### Participants and recruitment

The survey was completed by self-identified mental health practitioners working with infants, children, young people, and/or their families in Australia to deliver mental health or wellbeing services. Recruitment involved outreach to a range of government and non-government child and family services around Australia, primarily via email, social media posts, and flyers. Advertisements directed interested participants to the participation information sheet, consent form, and online survey hosted on REDCap between August 2022 and March 2023. Written informed consent was sought prior to survey completion. Along with questions about waitlists, drop-outs, barriers, and satisfaction levels, the survey also included questions about measure use, which are reported elsewhere (Tully et al., 2024).

### Measures

The survey was a combination of quantitative rating scales and qualitative open-response items. Questions included in the survey were selected through a literature review and input from a team of psychologists and researchers with expertise in CYMH. The draft survey was pilot tested with a convenience sample of practitioners, and items were subsequently revised to improve clarity before inclusion in the final survey.

### Practitioner characteristics

Demographic and practice information were collected from participants, including how often they work with parents in their service or workplace on a 5-point Likert scale ranging from *never* (1) to *always* (5). Practitioners who rated 3 or less were asked to indicate all their reasons for not working with parents more often from a list of reasons.

### Waitlists and waitlist management

Practitioners were asked whether they currently have a waitlist, and if so, what the average current wait times were at their service or workplace. Respondents indicated (via free response) the average current wait time and clear responses were recoded into categories (one month or less, more than one month to 3 months, more than 3 months to six months, more than 6 months to 12 months, more than 12 months, books closed, variable, and don’t know). Where respondents indicated a large range for waitlist times or provided two waitlist times across different places of work, the average was taken. Practitioners were also provided with a list of approaches for waitlist management and asked to endorse all that applied.

### Drop-outs

Practitioners were asked whether they keep track of the number of clients who drop-out. “Drop-out” was defined when the child, young person, or family ends treatment before service goals are met or fails to attend sessions even when followed up. Practitioners were asked to provide free responses for why they did not track drop-outs, and for those who did track drop outs, were asked about the reason why dropped out occurred. Responses were coded into categories and themes based on specific reasons reported.

### Perceived barriers to accessing care

Practitioners were asked to choose at least one and up to three barriers from a list of 13 barriers they perceive for children, young people and families accessing mental health and wellbeing support, with the list derived from the second Australian Child and Adolescent Survey of Mental Health and Wellbeing (Lawrence et al., 2016).

### Practitioner attitudes and perspectives

Practitioners were asked to rate their satisfaction with the current CYMH system in meeting the mental health support needs of children, young people, and families on a 5-point Likert scale ranging from *very unsatisfied* (1) to *very satisfied* (5). Practitioners had an optional free response question to explain further or provide reasons for their rating. Finally, practitioners were asked to rate the ease with which they could navigate other child, youth, or family mental health or wellbeing services (i.e., how difficult or easy is it for them to find referral options to recommend to their clients) on a 5-point Likert scale ranging from *very difficult* (1) to *very easy* (5).

### Data analysis

Quantitative variables were analysed using frequencies and descriptives in SPSS. Cases with missing data were included in analyses if demographic and professional data were complete and participants had answered at least approximately one-third of the survey questions. Qualitative data was exported to spreadsheets, and coded and analysed under a general inductive approach (Elo & Kyngäs, 2008), with a pragmatic orientation (Patton, 2014). Four members of the study team (JK, TN, OL, LG) coded the data adopting inductive content or thematic analysis (Braun & Clarke, 2006). The researchers engaged in regular discussions with one another throughout. Extracted themes were discussed and validated by all members of the research team and were presented back to the project operations team for member checking, with the operations team consisting of child and youth mental health researchers, practitioners, and clinician-researchers. Qualitative researcher positioning statements for JK, LG, OL, and TN can be found in Supplementary Material A.

## Results

### Practitioner characteristics

Of the 217 practitioners who began the survey, 206 completed at least one of third of the survey and were included in the study. The demographic, professional, and practice characteristics of respondents are shown in Table 1 and in the supplementary information. The sample included practitioners from all Australian states and territories (except Tasmania as no practitioners based there responded), but the majority worked in NSW (61.2%) and were based in metropolitan areas (68%). There was a range of reported practice settings, presentations practitioners worked with, and types of services provided. Practitioners mostly provided individual treatment for children and young people (91.7%; see Supplementary information) and many provided individual treatment to parents (76.7%). The types of services provided least commonly was dyadic work (e.g., parent-infant therapy; 17.5%) and group treatment for parents (4.9%).

**Table 1.** Demographic, professional, and practice characteristics of practitioners.

Demographic characteristics	Frequency	%
Gender		
Female	180	87.4
Male	25	12.1
Non-binary	1	0.5
Highest level of education		
Postgraduate university degree	133	64.6
Doctorate	47	22.8
Undergraduate university degree	26	12.6
Location		
Metropolitan	140	68
Regional	66	32
State or territory		
NSW	126	61.2
QLD	32	15.5
VIC	25	12.1
WA	18	8.7
SA	2	1
NT	2	1
ACT	1	0.5
**Professional characteristics**		
Profession*		
Psychologist**	145	70.7
Social worker	22	10.7
Nurse	17	8.3
Counsellor	4	1.9
Psychiatrist	3	1.5
Case worker	2	1.0
Occupational therapist	1	0.5
School counsellor	1	0.5
Other***	11	11.7)
**Practice characteristics**		
Practice setting/location*		
Public	101	49
Private practice	100	48.5
School	26	12.6
University	19	9.2
Non-government organisation	8	3.9
Other****	7	3.4
Child and youth age groups working with*		
13–18 years	181	87.9
3–12 years	154	74.8
19–25 years	109	52.9
0–2 years	59	28.6
Only work with parents		3.4
Frequency of working with parents in service or workplace		
Often	93	45.1
Always	87	42.2
Sometimes	20	9.7
Rarely	5	2.4
Never	1	0.5

*Denotes items where participants could choose more than one response; **Types of psychologists who responded: Clinical Psychologist, General Psychologist, Educational and Developmental Psychologist, Provisional Psychologist; *** Other professions: Art therapist; Clinical neuropsychologist; mental health dietician; peer support worker; physiotherapist; research psychologist; exercise physiologist; sport and exercise psychologist; specialist school principal; ****Other work settings: client’s residence, out of home care.

Most participants reported working with parents either *often* (45.1%) or *always* (42.2%) in their service or workplace. Of the 26 participants (12.6%) who reported that they *sometimes, rarely, or never work* with parents, common reasons indicated for not working with parents more often were service restraints (46.2%), time constraints (34.6%), parent involvement not necessary for the support provided (30.8%), and preference for working with the child or young person (30.8%). Less common reasons for not working with parents more often were lack of funding or government support (7.7%) and lack of confidence (7.7%). Other reasons indicated by participants were the young person’s decision and parents not being involved in the child’s care.

### Waitlists and waitlist management

Over half of respondents (*n* = 112; 54.4%) indicated their service or workplace currently had a waitlist. Table 2 displays the frequency and percentage of respondents who then reported waitlists of different durations.

**Table 2.** Current reported waitlist. *Frequency and percentage of reported length of current waitlist*

Length of current waitlist	Frequency	Percent
One month or less	22	21.6
More than 1 month to 3 months	33	32.4
More than 3 months to 6 months	22	21.6
More than 6 months to 12 months	10	9.8
More than 12 months	4	3.9
Books closed	2	1.9
Variable	5	4.9
Don’t know	4	3.9

*N* = 102.

Respondents were asked to endorse all the strategies that their services used to manage the waitlist if/when the service had a waitlist. In total, 83 of the 206 respondents indicated that their service has never kept a waitlist, so responses are based on 123 respondents. Of those, 60.9% provided recommendations for alternative face-to-face services, 43.9% provided information or resources to those on the waitlist, 41.4% provided options for online programs, 40.6% contacted the family or young person periodically to check-in and monitor, and 39.8% indicated that they only contacted the family when the family or young person moved to the top of the waitlist to offer a service.

### Monitoring drop-out

Just over half of respondents (108; 52.4%) indicated they keep track of the number of clients who drop-out from their service or program. Of those who indicated they do not keep track of dropouts, 62 respondents provided a response explaining why, with the most common reasons being time, resources, and the high administrative burden; it is not required by the service; because they have few drop-outs; and not having a system to track drop-outs (see Supplementary Material). Of those who indicated their service keeps track of drop-outs, 69.4% reported that they also keep track of the reasons why families or young people drop-out. Due to respondents often listing multiple reasons in their responses, reasons were coded into themes, with these being cost, time restraints for the family, lack of engagement and treatment readiness, and the family moving away.

### Perceived barriers to accessing support

The number and percentages for the main barriers that respondents endorsed for children, young people and families receiving support are displayed in Table 3. The top barrier endorsed by 60.6% of respondents was affordability of the service, followed by long waitlists (59.7%) and the child, young person, or parent refusing help or not turning up to the service (46.1%). Lack of skilled clinicians/services for certain presentations or complex needs was a barrier endorsed by 41.3% of practitioners. In terms of “other” barriers reported in open ended text responses, examples of these included difficulty accessing mental health support due to a complex mental health system, and feeling they have a limited number of Medicare-supported sessions, particularly for parents.

**Table 3.** Top three endorsed barriers to accessing support.

Perceived barriers	Frequency	Percent
Affordability	125	60.6
Long waitlists	123	59.7
Child/young person/parent refusing help/not turning up	95	46.1
Lack of skilled clinicians/services for certain presentations or complex needs	85	41.3
High severity thresholds to access certain services	80	38.8
Family/young person are not sure if help needed	79	38.3
Lack of service options in the area	75	36.4
Stigma around mental health or help-seeking	74	35.9
Parent/young person believes the problem will resolve by itself	67	32.5
Family/young person are not sure where to get help	65	31.5
Transportation	49	23.7
Prefer to handle the problem by themselves/family or friends	38	18.4
Other	13	6.3

*N* = 206.

### Perceived satisfaction and ease of navigation

Approximately 57% reported finding it difficult (46.6% *quite difficult* and 13.2% *very difficult*) to navigate child, youth, or family mental health or well-being services and find referral options in their clinical work. Around 27.9% were *neutral* in response to this question. Only about 15% found it easy to navigate the current CYMH system (13.2% *quite easy* and 2% *very easy*).

When asked about levels of satisfaction with the current CYMH system in meeting the needs of children, young people, and families, nearly 70% of practitioners indicated they were unsatisfied (30.4% were *very unsatisfied* and 39.2% were *somewhat unsatisfied*). A smaller proportion indicated they felt *neutral* (15.2%) and *somewhat satisfied* (15.2%). No participants indicated they were *very satisfied* with the current system. A total of 160 practitioners provided responses when asked to expand on their reasons for being satisfied or unsatisfied with the current CYMH system. Analysis of these responses led to four key themes which are presented and summarised below (Table 4). Some quotes have been shortened for space without changing the meaning. This is represented by an ellipsis (…). Consistent with the levels of satisfaction reported, most quotes presented describe dissatisfaction with the current system.

**Table 4.** Practitioner reasons for satisfaction or dissatisfaction with the current system.

Themes and subthemes	Quotes
**Service Funding**	
Public services underfunded	‘Government services are limited, underfunded … in the NT. The complexities of working with intergenerational trauma etc is not fully understood. Child protection are not coping and more needs to be done from a preventative perspective rather than a bandaid approach’. (NT Metro, Psychologist, 264) ‘Most funding is directed either to the private sector (in Medicare subsidies) or Headspace. CAMHS teams appears terribly underfunded, with new positions rare despite major growth in their target populations’. (NSW Metro, Psychologist, 114)
Gaps in funding	‘Where NDIS and Medicare do not overlap is a huge problem for Autistic clients. Medicare technically cannot be used for Autistic treatment as it is a disability, but NDIS won’t explicitly fund psychology in most people’s plans… Medicare has only just changed to let me see parents in relation to child treatment. Family therapy is not funded despite it being conclusively supported by evidence as being effective for treatment for ADHD, eating disorders, etc’. (VIC Regional, Psychologist, 267) ‘Private practice is limited by the Medicare number of sessions and rebate allowed and the admin/red tape takes up valuable time and money that could be better used in treatment. Assessment is not funded under Medicare in private despite developmental & learning issues being part of a MH presentation … [this] often means the comprehensive assessment is not comprehensive and treatment [is] compromised’ (QLD Metro, Psychologist, 269)
**Staffing**	
Training	‘Clinical Psychology graduates have usually had limited teaching in child trauma or child development or attachment or family therapy or play therapy (because its not considered to be evidenced based) and are trying to extrapolate from adult training. They no longer have access to on-the-spot training or support from more experienced clinicians and they’re not supported by the system so they’re at high risk of burnout and are likely to opt to work with children or youth privately’. (QLD Metro, Psychologist, 206)
Vacancies	‘There are so many staff vacancies across all levels of services and services are so overwhelmed in addition to the surge in numbers of young people seeking help that service provision is poor to non-existent’. (NSW Regional, Nurse, 93)
Burnout	‘Clinicians are doing the best they can given the resources and time they have. They are always doing more to try and help our young people, but this is eventually going to lead to burnout. We need many more skilled clinicians … ’. (NSW Regional, Psychologist, 160) ‘There is such a shortage of resources, professionals, and systems for referral. We are looking burnt out and have huge caseloads, the ‘system’ is non-existent’. (VIC Regional, Psychologist, 210)
**Availability/Accessibility**	
Waitlists	‘It’s very difficult for young people/families to access mental health services. The waitlists are long, services bounce the family around between other services for many reasons and families end up giving up and disengaging’ (QLD Metro, Social worker, 142)
Limited services and supports	‘Limited low-cost services for low to moderate severity, long waitlists in private practice, limited services addressing concurrent disability and mental health, limited availability of child and adolescent psychiatrists’. (NSW Metro, Psychologist, 10) ‘Lack of mental health professionals (e.g., paediatricians, paediatric psychiatrists, clinical psychologists who specialised in ADHD, autism or eating disorders) available in the area (i.e., long wait-list and/or facing with very high cost if go with private practice) has caused so much delay for children and families to receive help and support that they desperately need’. (WA Metro, Research psychologist, 211)
Lack of services for more complex presentations	‘There is a significant gap for moderate level severity outpatient support. There is no mental health inpatient service for young people in our regional area (0–18) and the adult service is not appropriate for adolescents (up to 25). There needs to be greater support (e.g., more YPARC) as well as outreach services/in-home MH support (by trained MH clinicians) for those who slip through the gaps. Coming into a clinic is not a good fit for most young people and leads to drop-out … There needs to be more in schools, at sport clubs, readily accessible and affordable’. (VIC Regional, Psychologist, 53)
Cost to families	‘I don’t think it’s affordable for many families. If the kid is not actively suicidal, they’re not likely to get CAMHS support so they need to go private – that means a long wait list and expenses’. (SA Metro, Psychologist, 202) ‘Lots of the system works well but I think there can be a lack of free good quality services for families’. (NSW Metro, Psychologist, 544)
**Fragmentation and unclear pathways**	‘Little shared information between different parts of the sector if more than one service involved, lack of coordination for those accessing treatment from multiple agencies, parents having difficulty navigating what is on offer’. (QLD Metro, Psychologist, 90) ‘I think a lot of individuals in a range of public and private services are trying very hard to meet the needs. There is severe fragmentation between the services and a lack of clear lines of referral’. (QLD Metro, Psychologist, 246) ‘Poor coordination, systematic mapping. … confusing system (not really a system. System isn’t clear for clients; difficult to navigate)’. (NSW Metro, Counsellor, 223)

#### Theme 1: service funding

The primary funding concern reported by practitioners related to a lack of publicly funded/free services. Practitioners in the study also raised concerns around funding gaps, for example with the current Medicare Benefits Schedule and National Disability Insurance Scheme (NDIS) in meeting the needs of children and youth with neurodevelopmental diagnoses, such as ADHD and Autism Spectrum Disorder (ASD). Generally, practitioners also highlighted gaps in what is covered by Medicare rebates, such as cognitive assessments and family therapy, leading to a lack of comprehensive assessment and treatment. Some practitioners also viewed the current ten psychology sessions subsided by Medicare as insufficient and found the previous 20 sessions available during the height of the COVID-19 pandemic being far better for providing adequate support and intervention.

#### Theme 2: staffing

Several concerns around staffing and the CYMH workforce were reported and broadly related to a perceived lack of training for staff, burnout, and vacancies in services. Specific concerns raised by many practitioners were around a lack of child and adolescent psychiatrists, and not enough practitioners trained to manage complex clinical cases and families. Practitioners believed the training provided by universities and higher education institutions to be insufficient, such that psychologists are being primarily taught adult models of care and lack specific training on topics needed for CYMH work such as attachment, child development, and family therapy. Finally, concerns about practitioner burnout reported were related to the under resourced system (mentioned above), high workloads for current staff, and insufficient supervision and support from experienced practitioners at workplaces.

#### Theme 3: availability and accessibility

The availability and accessibility of services was a key system issue contributing to dissatisfaction experienced by practitioners. Long waitlists to access psychology and psychiatry appointments across public and private services was commonly highlighted as a primary concern. A related issue contributing to long waitlists, was an overall under-resourced system and a limited number of mental health and wellbeing services (both assessment and treatment) for children, young people, and families. Many practitioners highlighted there are not enough government or public services providing family-based support. A common experience for practitioners is that the “stretched” public system (primarily Child Adolescent Mental Health Services) typically only has capacity to see young people who are acutely suicidal and cannot support longer term work that would lead to more lasting change. Practitioners also highlighted the cost of accessing private services is prohibitive for many families. Unsurprisingly, practitioners from regional or rural areas reported a significant lack of services across both public and private sectors, with examples including that services are over an hour away, or there is only one private practitioner in their town. Some practitioners acknowledged that there are good services that are trying their best to meet the needs of clients, but again, there are not enough to meet demand.

#### Theme 4: fragmentation and unclear pathways

Practitioners reported dissatisfaction with the structure and organisation of services within the CYMH system. A lack of coordination and communication between services was identified, which was problematic particularly when multiple agencies were providing support to a child, young person, or family. Unclear lines of referral were also identified as issues by practitioners, and many noted that parents are finding it difficult to navigate what services are available.

## Discussion

This study is the first to our knowledge to collect both quantitative and qualitative survey data to understand the current practices and perspectives of a diverse sample of Australian CYMH practitioners. Practitioners provided direct commentary on the current system and almost 70% of respondents reported being dissatisfied with the current CYMH system in meeting the needs of children, young people, and families. No practitioners reported feeling very satisfied with the current system. Over half (57%) of practitioners in our study reported finding it hard to navigate the current system and find referral options. One cannot help but wonder, if practitioners working in the system find it hard to navigate, how hard must it be for young people and families? Indeed, the recent Australian Child and Adolescent Survey of Mental Health and Wellbeing suggests service navigation is a great challenge, with the most commonly identified reason for parents of 4–17-year-olds not seeking or receiving more help was “not being sure where to get help”.

In terms of key barriers to accessing care, we also found shared perspectives between surveyed practitioners and previous parent reports, highlighting critical targets for change. In the aforementioned Australian Child and Adolescent Survey of Mental Health and Wellbeing (Lawrence et al., 2015), affordability (“Not being able to afford help”) was the second highest barrier endorsed by parents of children aged 4–17 years old, and waitlists or closed books (“Couldn’t get an appointment”) was the third most common barrier that parents identified. Similarly, the top barrier endorsed by respondents in our study was affordability of the service (60.6%), followed by long waitlists (59.7%). Although the barriers reported here are practitioner perspectives and not confirmed by children, youth and/or parents, practitioners “on the ground” would have awareness of, and at times directly witness, barriers faced by the clients they work with. Across other items and responses in the current survey, the issue of waitlists, cost, accessibility, and time were glaring. For example, under half of respondents indicated they do not keep track of dropouts, and common reasons for not tracking this data were a lack of time and resources. Of those who track dropouts, reported reasons for why clients have dropped out from services included cost and time restraints for the family.

In terms of waitlists, about half of our practitioner sample reported their service currently had a waitlist and reported a wide range of wait times, with the most common reported to be one and three months, followed equally by three to six months, and one month or less. Just under 10% had a waitlist that was six-to-twelve months. A minority reported a waitlist longer than 12 months, or that their books were closed. Of note, while half of respondents indicated they did not have a waitlist, our survey did not specifically ask reasons why they did not have a waitlist, and so it cannot be assumed these respondents were able to see clients straight away or that their books were not closed. As such, the current percentage reported of those who do and do not keep a waitlist should be interpreted with caution. Lengthy waitlists were similarly reported by Mulraney et al. (2021), who found average wait times for psychiatrists and psychologists in two states (VIC and SA) were 41 and 34 days, respectively. While direct comparison cannot be made due to methodological differences in the respective studies, both findings suggest many families are not able to access a service at the time of need and this issue is not improving over time. In addition to increased government funding of services, current waitlist data suggests a need for growth and innovation in service delivery in Australia. Indeed, our group (Growing Minds Australia), Australia’s first clinical trials network for CYMH (Hawes et al., 2023), is focusing on developing stepped-care models inspired by the United Kingdom’s “Improving Access to Psychological Treatments (IAPT)” program (Clark, 2011, 2018; Wakefield et al., 2021), as well as trialling and implementing a nation-wide, online, and streamlined assessment and triage system with appropriate evidence-based pathways to care.

Qualitative responses in our survey highlighted areas of dissatisfaction experienced by practitioners and can be broadly categorised into service factors (availability/accessibility, underfunding, fragmentation) and workforce factors (staff vacancies, burnout, and insufficient training), which were two key barriers also reported in Paton’s et al. (2021) clinician interviews. These themes show the interacting layers and difficulties faced by practitioners in the current system. Indeed, underfunded services (Theme 1) and difficulties with staffing (Theme 2) are likely underlying causes for problems with availability and accessibility (Theme 3). Burnout and staff vacancies, particularly at public or low-cost services, likely exacerbate the lack of availability and high thresholds to access these services as there is insufficient staff to provide support. High levels of dissatisfaction reported by practitioners risks continued staff turnover, which would further reduce the available workforce and services. Long waitlists and fragmentation in the system present a difficult combination of factors to practitioners – as young people and families are turned away or placed on waitlists by services, they are faced with a challenging system to navigate when finding alternative services. Without expanding and improving training and workforce development, issues around staff vacancies will likely remain unchanged, and it is unclear whether children, young people, and families will be provided with suitable, evidence-based care once they eventually access a service.

The findings reveal several policy, research, and practice implications regarding improving the current CYMH system (see Table 5). For example, results showed most practitioners (87%) work with parents often. This is a positive finding given that interventions involving parents are more effective in treating child mental health symptoms than interventions that do not involve parents (Dowell & Ogles, 2010; Line et al., 2024); however, the current Medicare Benefit Schedule prohibits regular parent involvement and the delivery of parenting programs as only up to two Medicare-subsidised services per calendar year are allowed. Indeed, core funding systems in Australia need to better align with research and practitioner desires to work wholistically with children and young people. Other key solutions leading from the results include increased funding of public services, expanding the CYMH workforce with targeted and specialised training, and improved organisational support to proactively manage burnout and so practitioners want to stay in publicly funded services. Several practitioner-informed ideas for enablers of change are also outlined by Paton et al. (2021), and further research into technology-enhanced training (Stylianakis et al., 2023) and service delivery (Chen et al., 2024; Georgeson et al., 2020; Jones, 2018) may also address significant gaps in care.

**Table 5.** Key findings and recommendations.

Key finding	Recommendations for research, practice, and policy
Most practitioners worked with children and young people 3–18 years old. Less than one third worked with infants and children under 3 years old.	Explore barriers and enablers for practitioners working with infants and very young children. Expanding workforce capability to work in early intervention (e.g., through training).
Many practitioners (89%) in Australia are working with parents or include parents in their work.	Future research on the specific parenting interventions delivered by services in Australia. Ensure practice is evidence-based and linked to core competencies working with parents and families. Government funding to align with best practice (i.e., Medicare Benefits Schedule expanding to fund full programs of parent-only treatment).
Half of services have a waitlist.	Implementation of stepped-care models to improve access to timely support. Innovative online programs to provide immediate assessment and evidence-based recommendations.
Underfunded services and high costs to families are barriers to care.	Increased government funding for public services. Increase in number of subsidised Medicare sessions.
Inadequate training in CYMH and staff burnout a concern.	University programs providing increased training on infant child youth mental health and associated frameworks. Organisational support for supervision and mentorship of younger clinicians. Organisational support for staff training.
Fragmentation in the mental health system.	A centralised, online system for practitioners, young people, and families to receive recommendations for services in their local area.

The strength of this study was the diverse range of practitioners, represented by over 10 different types of professions across many settings and supporting a variety of mental health presentations. However, there are several limitations that should be considered in the current study. While attempts were made to sample practitioners across Australia and there was at least some representation from all states and territories (except one state), most participants were from two of the largest states (NSW and VIC). Although these are the most populous states, national representation could have been strengthened by increasing our sample size in other states and territories. As the survey was voluntary, it is possible respondents were more motivated to participate as they had particularly negative experiences with the current mental health system. Finally, as this was a self-report survey, reports on certain items, such as how often practitioners worked with parents and current wait times to access services were not substantiated and would require a full-service evaluation in future. Due to the relatively small sample size, future research of this kind would be strengthened by a larger sample of practitioners and allow for additional analyses to be conducted, such as associations between variables of interest (e.g., are satisfaction levels with the current system influenced by practitioner location?). Further, future surveys would benefit from asking practitioners about their perspectives on specific strengths in the system and what is working well in order to highlight aspects of the system that can be enhanced and fostered.

Overall, this study extends on findings from previous research by including a broad range of professionals delivering mental health and wellbeing services to children, young people, and families. We also sought additional practice information on issues, such as current waitlists, waitlist management strategies, and treatment dropouts. Together with previous studies, the current findings aim to further highlight the voices of practitioners and their current challenges working within the system, and we call on the government to prioritise improved provision of support for children and young people with mental health difficulties in Australia.

## Supplementary Material

Practitioner Perspectives CYMH Supplementary Material

## Data Availability

The data that support the findings of this study are available on request from the corresponding author, JK. The data are not publicly available due to their containing information that could compromise the privacy of research participants.
