# Supplementary material for: Practitioner perspectives on the child and youth mental health system in Australia: what needs to change?
Source: Aust J Psychol. 2025 Jun 4;77(1):2509649. doi: 10.1080/00049530.2025.2509649 (PMC12218569; doi:10.1080/00049530.2025.2509649)
Supplement: Practitioner Perspectives CYMH Supplementary Material [file RAUP_A_2509649_SM5227.docx]

**Supplementary A – Qualitive researcher positioning statements**

JK

This researcher is a clinical psychologist and university lecturer with current clinical experience in early intervention, perinatal infant mental health, and parenting support.

TN

This researcher has a background investigating the limits of quantitative research methods, and is a registered psychologist in practice with experience in community psychology settings, and with a psychodynamic and post-structuralist orientation.

OL

This researcher is an early career clinical psychologist. She has lived familiarity with the participant group, having previously worked in a child and youth community mental health service (public health). Her background is primarily in quantitative research methods.

LG

This researcher has a background in human rights and social justice research, with a Masters in Peace and Conflict Studies and grassroots experience working in the community sector with vulnerable young people in remote and regional areas.

**Supplementary B – Additional practice characteristics reported by practitioners**

| **Practice characteristics** | **Frequency** | **%** |
| --- | --- | --- |
| Clinical presentations working with* |  |  |
| Teen and youth anxiety | 167 | 81.1 |
| Teen and youth depression | 162 | 78.6 |
| ADHD | 146 | 70.9 |
| Child and youth behavioural problems | 145 | 70.4 |
| Child anxiety | 138 | 67 |
| Autism Spectrum Disorder | 131 | 63.6 |
| Eating Disorders | 91 | 44.2 |
| Early psychosis | 77 | 37.4 |
| Intellectual disability | 69 | 33.5 |
| Alcohol and substance use | 67 | 32.5 |
| Perinatal infant mental health | 29 | 14.1 |
| Work only with parents | 5 | 2.4 |
| Other** | 32 | 15.5 |
| Type of services providing* |  |  |
| Individual treatment/support for child or young person | 189 | 91.7 |
| Individual treatment/support for parents | 158 | 76.7 |
| Family therapy | 80 | 38.8 |
| Case management | 76 | 36.9 |
| Group treatment/support for children or young people | 65 | 31.6 |
| Dyadic work (e.g., parent-infant therapy) | 36 | 17.5 |
| Group treatment/support for parents | 39 | 18.9 |
| Other*** | 10 | 4.9 |

*Denotes items where participants could choose more than one response; **Other presentations working with: complex trauma, posttraumatic stress disorder, suicidality and self-harm, chronic medical illness, functional neurological disorder, personality disorders, sexual assault, child protection, family separation, bipolar disorder; ***Other services: neuropsychological assessment, acute mental health assessment, consultation liaison.

**Supplementary C – Practitioner reported reasons for not keeping track of drop-outs**

| **Reason** | **Frequency** | **Percent** |
| --- | --- | --- |
| Time, resources, and administrative burden | 19 | 30.6 |
| Not required by service* | 17 | 27.4 |
| Few drop-outs | 8 | 12.9 |
| No systems in place | 7 | 11.3 |
| Managed elsewhere** | 6 | 9.7 |
| Don’t know | 4 | 6.5 |
| Unsure why tracking is needed | 1 | 1.6 |

*Some responses pertained to practitioners working in an acute setting; **Pertained to reports of either administration team or managers keeping records of drop-outs at the service.
